# Genomic prediction and selection response for grain yield in safflower

**DOI:** 10.3389/fgene.2023.1129433

**Published:** 2023-03-27

**Authors:** Huanhuan Zhao, Zibei Lin, Majid Khansefid, Josquin F. Tibbits, Matthew J. Hayden

**Affiliations:** ^1^ School of Applied Systems Biology, La Trobe University, Bundoora, VIC, Australia; ^2^ Agriculture Victoria, AgriBio, Centre for AgriBioscience, Bundoora, VIC, Australia

**Keywords:** genomic selection, safflower, multivariate, selection response, grain yield

## Abstract

In plant breeding programs, multiple traits are recorded in each trial, and the traits are often correlated. Correlated traits can be incorporated into genomic selection models, especially for traits with low heritability, to improve prediction accuracy. In this study, we investigated the genetic correlation between important agronomic traits in safflower. We observed the moderate genetic correlations between grain yield (GY) and plant height (PH, 0.272–0.531), and low correlations between grain yield and days to flowering (DF, −0.157–0.201). A 4%–20% prediction accuracy improvement for grain yield was achieved when plant height was included in both training and validation sets with multivariate models. We further explored the selection responses for grain yield by selecting the top 20% of lines based on different selection indices. Selection responses for grain yield varied across sites. Simultaneous selection for grain yield and seed oil content (OL) showed positive gains across all sites with equal weights for both grain yield and oil content. Combining g×E interaction into genomic selection (GS) led to more balanced selection responses across sites. In conclusion, genomic selection is a valuable breeding tool for breeding high grain yield, oil content, and highly adaptable safflower varieties.

## Introduction

Genomic selection (GS), as an efficient breeding tool, was first implemented in animal genetic improvements (Schaeffer, 2006; Meuwissen et al., 2013). With the availability of genome-wide markers and low-cost genotyping technologies, GS is rapidly adopted in plant genetics and breeding (Heffner et al., 2009; Lin et al., 2014). In GS, a training population, which has been genotyped and phenotyped, is used to train a statistical model to predict individuals that have been genotyped but not phenotyped. The predicted value is termed as an estimated breeding value (EBV) if the model utilized a pedigree relationship between individuals and a genomic estimated breeding value (GEBV) if marker data is used in the analysis. The EBVs or GEBVs can be used to rank and select germplasm (Meuwissen et al., 2001; de Los Campos et al., 2013).

Implementing GS in the breeding program heavily depends on the prediction accuracy of GEBVs. Multivariate models showed higher prediction accuracy than univariate models in GS studies (Jia and Jannink, 2012; Sun et al., 2019). The additional information in genetically correlated traits is exploited in multivariate models, and the higher the correlation is, the greater the multivariate models would benefit (Zhao et al., 2022a). Rutkoski et al. (2016) included canopy temperature and normalized difference vegetation index in a multivariate model, which resulted in a 70% prediction accuracy improvement for grain yield in wheat. Important agronomy traits, such as days to flowering, plant height, etc., with malting quality traits, were used to assess the prediction accuracy of multivariate models, and the result showed a 76% higher predictive ability than univariate models (Bhatta et al., 2020).

GS can improve the genetic gain of target traits in crop breeding programs mainly by increasing selection accuracy, shortening breeding cycles, etc. (Falconer and Mackay, 1996; Xu et al., 2017). Studies to compare genetic gains achieved by different selection strategies have been conducted with real datasets and simulations (Woolliams et al., 2015; Marulanda et al., 2016). Simulation with ryegrass showed a 4-year reduction in cycle time, with genetic gain doubling or tripling when GS is incorporated into the breeding program (Lin et al., 2016). In the short breeding cycle, improving the selection accuracy would be important for higher genetic gain. Beyene et al. (2015) estimated genetic gains in grain yield in eight bi-parental maize populations and observed higher grain yield achieved from GS than conventional pedigree-based phenotypic selection (PS). A study with wheat indicated that the integrated PS + GS approach could result in an optimal genetic gain for yield (Lozada et al., 2020).

GS can simultaneously select multiple traits with the genomic selection index (GSI), a linear combination of ‘traits’ GEBVs. Both simulation and real data showed that GSI was more efficient than the phenotypic selection index (PSI) per unit of time (Ceron-Rojas et al., 2015; Habyarimana et al., 2020b). A study with maize confirmed that GSI is more efficient in obtaining sustainable gain than the phenotypic selection index (PSI) per unit of time in selection for grain yield, plant height, etc. (Ceron-Rojas et al., 2015). Various GSI were compared in wheat, and the results indicated the employed GSI mitigated the negative trade-off between grain yield and protein content and led to a substantial selection response for protein yield (Michel et al., 2019). In multitrait index selection, the choice of the selection index and the assignment of the weights to different traits strongly affected the selection gain (Marulanda et al., 2021).

Safflower is a multi-purpose crop that is grown worldwide in Africa, America, Europe, and Asia (Zohary, 1999). It is grown as a vegetable, cut flower, herbal medicine, bird feed, etc. However, the main growing interest has shifted to safflower seed oil because of its high oleic and linoleic acid content (Li and Mündel, 1996; Khalid et al., 2017). In 2020, the global safflower growing area was about 0.8 million ha, and it was around 40,000 ha in Australia (FAO, 2020). Safflower has the potential to be further incorporated into the farming system because of its drought tolerance. Two Australian safflower varieties released in 1998 carried resistance to leaf spot (*Alternaria Carthami*) and root rot (*Macrophomina phaseolina*), respectively, and most varieties grown today in Australia were developed overseas (Jochinke et al., 2008). Breeding for high-yield safflower varieties is under demand. In this study, we attempted to evaluate the genetic gain by adopting genomic selection to select elite crossing parents from a diverse Genebank collection. The aims of the study were 1) to estimate the genetic correlation between major safflower agronomy traits; 2) to evaluate the genomic prediction accuracy for safflower grain yield by combining other traits with multivariate prediction models; 3) to compare selection responses of grain yield to different safflower selection strategies.

## Materials and methods

### Plant material, phenotyping, and genotyping

The plant material used in this study belongs to a diverse safflower collection sourced from the Australian Grain Genebank. The accession information, field design, and genotyping detail have been described previously (Zhao et al., 2021). Briefly, all accessions in the collection were tested in 2017 and 2018 with two trials each year: site 1 (2017 irrigation site, IR), site 2 (2017 rainfed, RF), site 3 (2018 rainfed in low rainfall zone, LR), and site 4 (2018 rainfed in high rainfall zone, HR). Site IR was the optimal site. Site RF suffered water stress at the flowering stage, and sites LR and HR suffered water stress during the whole safflower growing period. Field trials adopted a randomized complete block design with 2-3 replications, and the plot size was 1 m × 5 m with five rows in each plot. Eight traits were recorded (Supplementary Table S1), including days to flowering (DF, days from sowing to 25% of the plot flowering), days to maturity (DM, days from sowing to 90% of the plot being physiologically mature), flowering time (FT, days from flowering to mature), plant height (PH, in cm), seed weight (SW, Gram/per 500 achenes), grain yield (GY, t/ha, with the plot width 1.25 m), seed protein (PC%), and seed oil content (OL%).

A total of 349 safflower accessions were genotyped with genotyping-by-sequencing (GBS). Six seeds per accession were crushed, and genomic DNA was extracted, digested, amplified, purified, and sequenced with Illumina Hiseq 3,000 sequencer with in-house GBS protocols. SNPs were filtered with a missing data rate of <50% and minor allele frequency (MAF) > 0.01 for this study. The resulting 6,911 SNPs were imputed with LinkImpute (Money et al., 2015) and showed in Supplementary Table S2. Details on the population structure of those accessions have previously been described by Zhao et al. (2021).

### Genomic parameters estimation

A multivariate linear mixed model with the variance-covariance matrix was used to estimate the genetic parameters (i.e., heritability, genetic variance, and genetic correlation between traits) at each site. The model could be illustrated as follows:

$$\left[\begin{array}{c} y_{1}\\ y_{2} \end{array}\right]=\left[\begin{array}{cc} X_{1} & 0\\ 0 & X_{2} \end{array}\right]\left[\begin{array}{c} b_{1}\\ b_{2} \end{array}\right]+\left[\begin{array}{cc} Z_{g1} & 0\\ 0 & Z_{g2} \end{array}\right]\left[\begin{array}{c} g_{1}\\ g_{2} \end{array}\right]+\left[\begin{array}{cc} Z_{r1} & 0\\ 0 & Z_{r2} \end{array}\right]\left[\begin{array}{c} r_{1}\\ r_{2} \end{array}\right]+\left[\begin{array}{cc} Z_{c1} & 0\\ 0 & Z_{c2} \end{array}\right]\left[\begin{array}{c} c_{1}\\ c_{2} \end{array}\right]+\left[\begin{array}{c} \varepsilon_{1}\\ \varepsilon_{2} \end{array}\right]$$ (1)

where **y**_**1**_ and **y**_**2**_ are the vectors of two traits, **b**_**1**_ and **b**_**2**_ are the vectors of two traits’ mean and replications, **g**_**1**_ and **g**_**2**_ are the vectors of random genetic effects following a variance-covariance matrix of two traits, as $\left[\begin{array}{c} g_{1}\\ g_{2} \end{array}\right]∼N\left(0,I⊗T\right)$ in where $T=\left[\begin{array}{cc} \sigma_{g1}^{2} & \sigma_{g12}\\ \sigma_{g21} & \sigma_{g2}^{2} \end{array}\right]$ , **r**_**1**_ **, r**_**2**_, **c**_**1**_, and **c**_**2**_ are the field design row and column vectors of two traits, **ε**_**1**_ and **ε**_**2**_ are the residuals of two traits also following a variance-covariance matrix as $\left[\begin{array}{c} \varepsilon_{1}\\ \varepsilon_{2} \end{array}\right]∼N\left(0,I⊗R\right)$ , in where $R=\left[\begin{array}{cc} \sigma_{e1}^{2} & \sigma_{e12}\\ \sigma_{e21} & \sigma_{e2}^{2} \end{array}\right]$ . **X**, **Z**_**g**_, **Z**_**r**_, and **Z**_**c**_ are the incidence matrices associating phenotypes with fixed and random effects, and **I** is the identity matrix. Phenotypic variance $\left(\sigma_{P}^{2}\right)$ were calculated according to: $\sigma_{P}^{2}=\sigma_{G}^{2}+\sigma_{e}^{2}$ where $\sigma_{G}^{2}$ is the genetic variance, $\sigma_{e}^{2}$ is the residual variance. The broad sense heritability (H^2^) was calculated as follow:

$$H^{2}=\sigma_{G}^{2}/\left(\sigma_{G}^{2}+\sigma_{e}^{2}\right).$$ (2)

The phenotypic correlation between traits was calculated as:

rPx,y=covPxyσPx2σPy2, (3)

where *σ*_Px_^2^ and *σ*_Py_^2^ are the phenotypic variances of traits x and y, ${cov}_{P\left(xy\right)}$ is the covariance. When the genomic relation matrix (**G**) replaced I in the model, narrow sense heritability (h^2^) was calculated as the proportion of additive genetic variance $\left(\sigma_{A}^{2}\right)$ to the total phenotypic variance:

$$h^{2}=\sigma_{A}^{2}/\left(\sigma_{A}^{2}+\sigma_{e}^{2}\right)$$ (4)

The additive genetic correlation ( $r_{A\left(x,y\right)}$ ) was calculated the same as the phenotypic correlation but with the additive genetic variance (*σ*_Ax_^2^, *σ*_Ay_^2^) and covariance ( ${cov}_{A\left(xy\right)}$ ). All calculations were conducted with ASReml (Gilmour et al., 2015).

### Prediction accuracy for grain yield

The best linear unbiased estimates for each safflower accession (BLUEs) were estimated for each trial the same as the previous study (Zhao et al., 2021) by fitting accessions as fixed effects and an autoregressive (AR) correlation structure in the error to account for the spatial variation. GBLUP model was adopted for the genomic prediction accuracy evaluation. It could be illustrated as follow:

y=Xb+Zg+e (5)

where **y** is the vector of BLUEs, **b** is the vector of means, **g** is the vector of additive genetic effects, **e** is the residual, and **X** and **Z** are the correspondent design matrix for **g** and **b**. When the BLUEs of single trait GY (univariate), GY with PH/DF, and GY, PH, and DF (multivariate), were fitted respectively, different prediction models were developed for estimating the prediction accuracy of GY.

The prediction accuracy was defined as ‘Pearson’s correlation coefficient between GEBVs and BLUEs for GY by a five-fold cross-validation method. All accessions were randomly divided into five equal subsets. Each subset was, in turn, chosen as the validation set and was subsequently predicted by using the other four subsets as the training set. The prediction accuracies of multivariate models were compared from two cross-validation prediction scenarios. The first cross-validation scenario (MT-CV1) predicted accessions in the validation set that have been phenotyped with PH and DF but not GY, while the training set had phenotypes of GY, PH, and DF. The second cross-validation scenario (MT-CV2) predicted the performance of accessions in the validation set that have not been evaluated with GY, PH, and DF, while the training set had been phenotyped with GY, PH, and DF. The whole process was repeated five times. We calculated the mean prediction accuracy and standard deviation (SD) across all 25 validation sets at each site.

### Selection response

Aside from assessing the genomic prediction accuracy for GY, we further explored the selection responses of two breeding strategies with different indices, which 1) aimed to select high-yield genotypes and 2) to select simultaneously high-yielding and high-oil content genotypes. Zhao et al. (2022b) observed high correlations between performance of lines at the site LR and HR for all traits, and outcomes should be comparable between LR and HR. Therefore, we only calculated the selection responses at sites IR, RF, and LR.

For selecting GY, we used BLUEs as the phenotypic selection index to perform phenotypic selection (PS). GEBVs estimated from the univariate model as the first GS index (SGS) and GEBVs estimated from the multivariate model (PH and GY) as the second GS index (MGS) were used for the genomic selection (GS), respectively.

For simultaneously selecting GY and OL, we compared two indices. We gave equal weight to GY and OL without considering the correlation between those two traits, represented as multi-trait GS index 1 (MTGS1). The GEBVs estimated for GY and OL with univariate GBLUP models were standardized before applying the weights. The second multi-traits GS index (MTGS2) was calculated with unequal weights, calculated following the formula: $b=P^{-1}g,$ where **b** is a vector of index weight for each trait, **g** is a vector of the additive genetic variance of the traits, **P** ^−1^ is the inverse of the phenotypic variance-covariance matrix (Dekkers, 2007):

$$\left(\begin{array}{cc} \sigma_{p1}^{2} & \sigma_{p12}\\ \sigma_{p21} & \sigma_{p2}^{2} \end{array}\right),$$ (6)

where $\sigma_{p1}^{2}$ and $\sigma_{p2}^{2}$ are the phenotypic variance of GY and OL, and $\sigma_{p12}$ is the covariance. All the genomic parameters were estimated with model 1. MTGS1 and MTGS2 were calculated in a conventional way as the linear combination of the weighted GEBVs.

All selections with different indices were conducted at each site. The selection responses at the other two sites were also calculated with the same selected candidates to compare the response across sites. We further estimated GEBVs for GY and OL with a g×E GBLUP model, which combined three sites and described by (Zhao et al., 2021) as follows:

$$y=Xb+Zg+Z_{2}gE+e$$ (7)

Where terms are the same as in model (2), with gE being the interaction between site and additive genetic effects and **Z**_**2**_ being the incidence matrix. The combined GEBVs were used as GSI for GY selection (g×EGS) or combined with equal weights to simultaneously select for GY and OL (g×EMTGS1).

We selected the top 20% of the accessions based on indices as selected candidates. The selection differential (S) is calculated as the difference between the mean GY or OL of the selected candidates and the mean GY or OL of the diverse population at each site. We used the selection differential to calculate the selection response (R) according to R = h^2^S, where h^2^ is the narrow-sense heritability, and S is the selection differential. The percentage of the gain increased at each site was calculated to facilitate the comparison.

## Results

### Heritability and correlation

We estimated the variance components and the narrow sense heritability (h^2^) for all traits in each site. The results are averaged from pairwise traits combination and shown in Table 1. The estimated additive genetic variance (σ^2^_A_) was higher than the error variance (σ^2^_e_) for most traits at all sites, suggesting SNPs used in the study were able to capture the additive genetic variations in both the optimal site and stressed sites. SW and OL showed high estimated h^2^, ranging from 0.63 to 0.81, while PC, FT, and DM all showed moderate h^2^, around 0.5 at all sites. We observed large variations between sites for h^2^ for DF, PH, and GY. DF had a relatively low estimated h^2^ at site RF, 0.4, with high h^2^ at the other three sites. The estimated h^2^ for GY and PH were lower at sites LR and HR compared with sites IR and RF. The boxplot of BLUEs for each trait at each site was shown in Supplementary Figure S1.

**TABLE 1:** Estimated variance components and narrow sense heritability (h^2^) with SD in each site.

	IR			RF			LR			HR		
	σ^2^_e_	σ^2^_A_	h^2^ ± SD	σ^2^_e_	σ^2^_A_	h^2^ ± SD	σ^2^_e_	σ^2^_A_	h^2^ ± SD	σ^2^_e_	σ^2^_A_	h^2^ ± SD
SW	2.297	9.726	0.808 (0.0)	2.741	8.461	0.756 (0.001)	3.847	6.55	0.63 (0.001)	3.471	6.962	0.669 (0.002)
PH	61.84	176.286	0.74 (0.001)	52.116	152.129	0.745 (0.0)	55.524	23.94	0.301 (0.001)	41.729	35.894	0.463 (0.002)
DF	3.594	9.621	0.728 (0.003)	4.707	3.083	0.394 (0.003)	5.966	15.301	0.719 (0.0)	5.996	15.843	0.727 (0.002)
GY	0.557	0.682	0.548 (0.004)	0.291	0.294	0.502 (0.007)	0.11	0.034	0.229 (0.01)	0.089	0.037	0.287 (0.014)
OL	3.067	11.73	0.792 (0.001)	4.169	10.947	0.723 (0.001)	3.484	7.121	0.673 (0.003)	3.303	7.994	0.706 (0.001)
PC	0.294	0.342	0.539 (0.004)	0.272	0.395	0.592 (0.005)	0.26	0.327	0.559 (0.002)	0.38	0.483	0.559 (0.003)
FT	3.898	4.546	0.536 (0.004)	5.645	5.716	0.502 (0.003)	4.583	3.414	0.426 (0.002)	3.621	4.255	0.541 (0.002)
DM	6.163	13.534	0.686 (0.003)	2.583	4.028	0.609 (0.002)	12.844	12.696	0.498 (0.002)	7.274	9.503	0.567 (0.001)

Note: *σ^2^_e_, error variance; σ^2^_A_, additive genetic variance; ** IR, site 1 (2017 irrigation site), RF, site 2 (2017 rainfed), LR, site 3 (2018 rainfed in low rainfall zone), and HR, site 4 (2018 rainfed in high rainfall zone); *** DF, days to flowering; FT, flowering time; DM, days to maturity; PH, plant height; SW, seed weight; GY, grain yield; PC, seed protein content; and OL, seed oil content.

**FIGURE 1:**
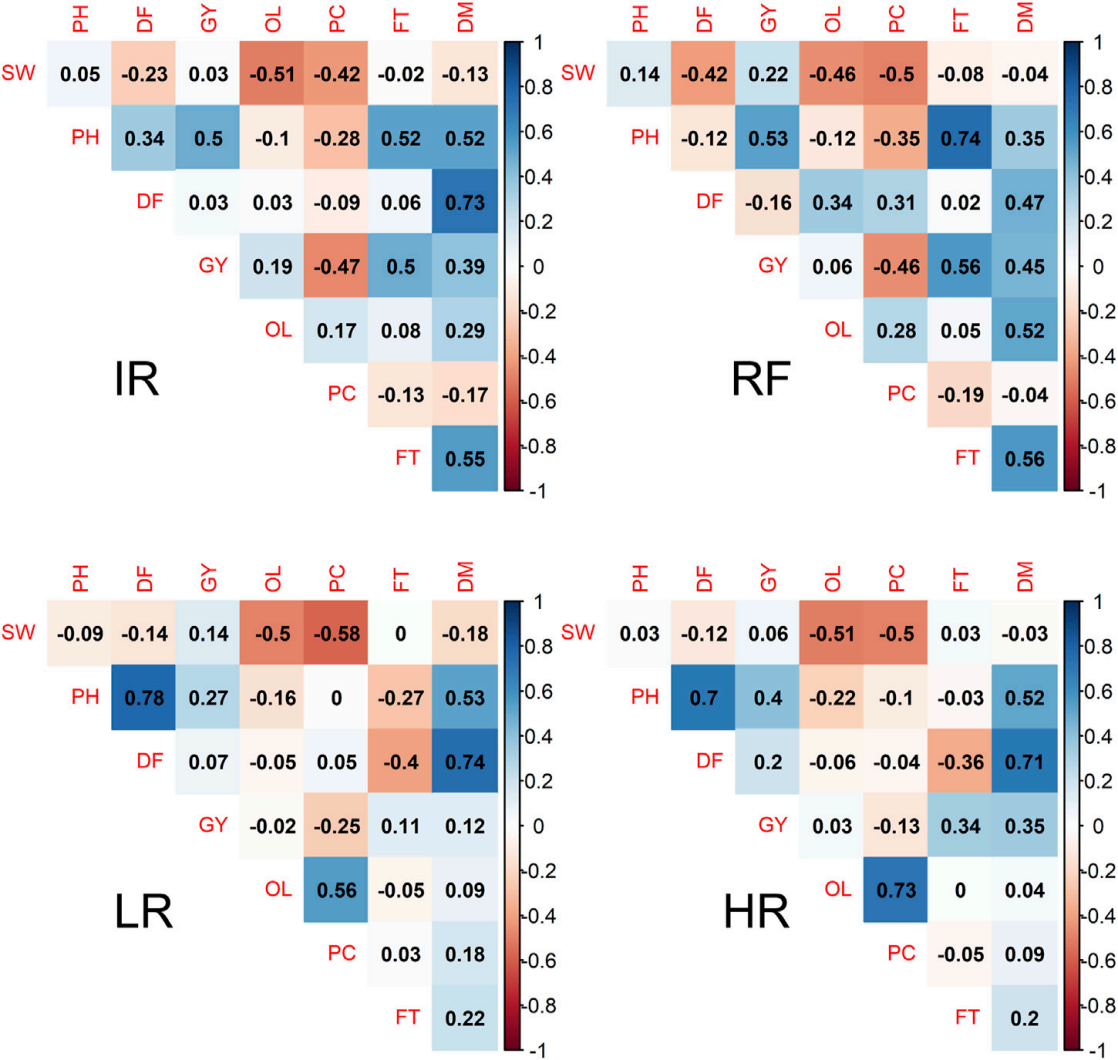
Additive genetic correlation among pairwise safflower traits in four field sites. Color ranged from dark orange to dake blue is correspondent to the genetic correlation (r_g_) from −1 to 1. The abbreviations used for sites and traits in this figure are described in Table 1.

The additive genetic correlations between traits within each site are shown in Figure 1. We observed the negative correlations between SW with OL and SW with PC at all sites (−0.4 ∼ −0.5), while OL and PC showed positive correlations with a strong positive correlation observed at sites LR (0.56) and HR (0.73). The correlations between OL and GY were low and varied across sites, from −0.02 at site LR to 0.19 at site IR. However, negative correlations between PC and GY were observed for all sites, ranging from −0.47 to −0.13. GY and PH showed a low positive correlation (0.27) at site LR, but it was moderate at the other three sites, and low correlations between GY and DF (−0.16–0.2) were observed at all sites. The phenotypic correlations between traits also showed similar patterns (Supplementary Figure S2).

### Prediction accuracy for multivariate models

**FIGURE 2:**
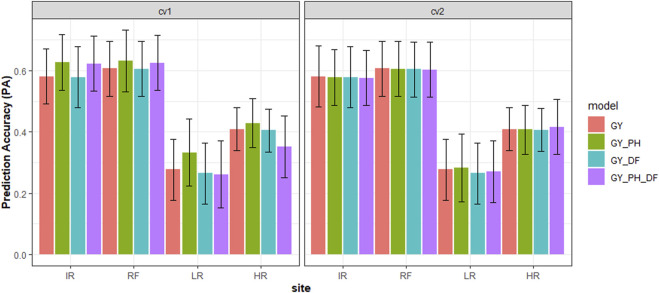
Cross-validation genomic prediction accuracy (PA) with standard deviation (black line) using different prediction models for grain yield (GY) in safflower with two scenarios at four sites. Note: * Scenarios = GY predicted in the validation set when the CV1: Had plant height (PH) and days to flowering (DF) phenotypes, or CV2: Had no PH and DF phenotypes.** Models = GY (univariate, GY), GY_PH (multivariate, GY and PH), GY_DF (multivariate GY and DF), and GY_PH_DF (multivariate, GY, PH, and DF) *** The abbreviations used for sites and traits in this figure are described under Table 1.

In this study, the grain yield (GY) at each site was predicted with univariate and multivariate models by fitting BLUEs as the “phenotypes”. All models showed higher prediction accuracy at sites IR and RF than at sites LR and HR in both scenarios (Figure 2). The prediction accuracy for grain yield varied across four sites with the highest accuracy achieved at site RF, 0.61, and lowest accuracy at site LR, 0.28 with the univariate model. In the CV1 scenario, we observed the prediction accuracy for the GY_DF multivariate model was comparable to univariate model. However, the GY_PH model showed higher prediction accuracy than the univariate model, with a 4% accuracy increase at site IR and a 20% increase at site LR. The GY_DF_PH multivariate model performed the same as the GY_PH multivariate model at sites IR and RF but with lower accuracy at sites LR and HR. In the CV2 scenario, we observed all multivariate models performed the same as the univariate model across all sites.

### Selection response to different selection strategies

**FIGURE 3:**
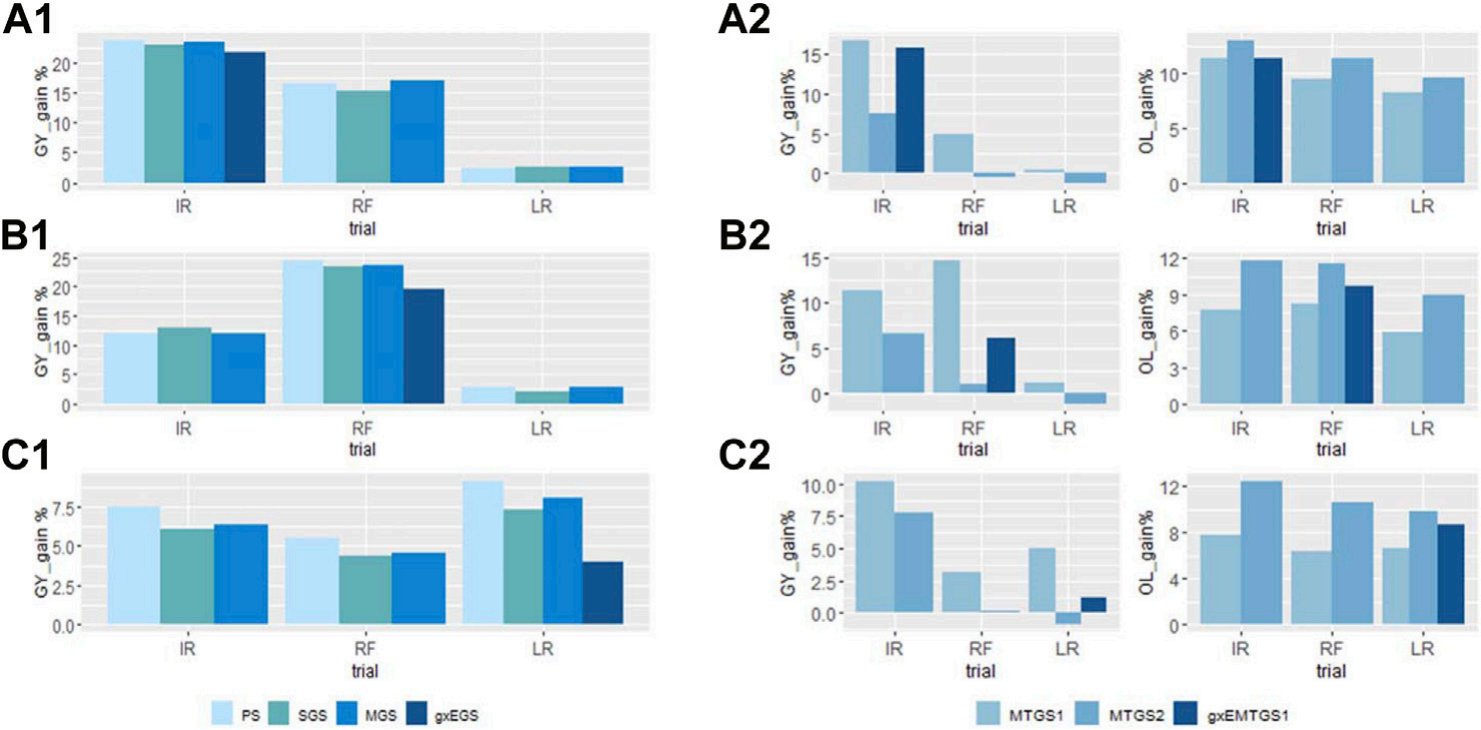
The percentage of the gain improved by using different selection indices for GY **(A1–C1)**, and GY and OL **(A2–C2)**. **(A1)** and **(A2)**: Selection conducted at site IR; **(B1)** and **(B2)**: Selection conducted at site RF; **(C1)** and **(C2)**: Selection conducted at site LR. PS, phenotypic selection; SGS, GS selection with GEBVs estimated with the univariate model; MGS, GS selection with GEBVs estimated with the multivariate model; g × EGS, GS selection with GEBVs estimated with sites combined g × E GBLUP model; MTGS1, GS selection with equal weighted GEBVs; MTGS2, GS selection with unequal weighted GEBVs; g × EMTGS1, GS selection with GEBVs estimated with sites combined g × E GBLUP model using MTGS1 selection strategy. The abbreviations used for sites and traits in this figure are described in Table 1.

The percentage of gain increase for each site was plotted in Figure 3. For the single trait GY selection (Figures 3A1–C1), we observed that the highest selection response for GY was achieved when the selection was conducted at the optimal site (IR), and the response was compromised at the stressed sites (RF and LR). When the selection was conducted at stressed sites, the selection response in the optimal sites was reduced dramatically (Supplementary Table S3). When selected at site IR, the yield gain was 0.7 t/ha, about a 23% yield increase. However, with the same selected candidate, the gain was compromised to 0.3 t/ha, about a 16% yield increase at site RF, and 0.03 t/ha, about a 2.6% yield increase at site LR. When selection at sites RF and LR, higher gains were achieved for both sites, with 0.4 t/ha, about 24% yield increase at site RF, and 0.08 t/ha, about 8% gain increase at site LR. But the gain for site IR was reduced to 0.36 t/ha (about 12% yield increase) and 0.2 t/ha (about 7% yield increase), respectively. With GEBVs predicted from the g×EGS model, we achieved a more balanced gain for the three sites, with 22% for IR, 20% for RF, and 4% for LR. The data revealed no major response differences between PS, SGS, and MGS for single traits GY selection, but MGS often performed a bit better than SGS. We didn't observe a big difference of gains between PS and GS when selecting at sites IR and RF, but the PS showed a slightly higher gain than GS when selecting at the LR site.

When the selection was performed for GY and OL simultaneously, the equal weight GS (MTGS1) and unequal weight GS (MTGS2) indices showed different gains for GY and OL (Figures 3A2–C2). MTGS1 method had all positive gains for GY and OL, while MTGS2 showed a higher gain for OL with negative gains for GY at RF and LR sites. When selecting at the site IR, the gain increase for GY varied dramatically across sites, from about 0.4% in LR to 16% in IR for MTGS1, with an 8.3%–11.4% gain increase for OL. MTGS2 showed a higher gain increase for OL, around 9.6–13.1-% OL, but negative responses for GY at RF and LR were observed when selected at the IR site. The combined g×EMTGS1 showed a 1.2%–15.8% gain for GY and an 8.7%–11.4% increase for OL (Figures 3A2–C2).

## Discussion

Safflower is an underutilized oil seed crop. Breeding efforts for genetic improvement of target traits were modest. With limited genetic and genomic resources and limited research funds, the datasets we used in our study have been used for genetic diversity study, genetic characterization, and genomic parameters estimation of important agronomic traits in safflower. All those studies enhanced our knowledge of safflower and paved the way to transfer conventional safflower breeding into a highly targeted and more efficient modern crop breeding scheme. In this study, we further investigated the genomic prediction accuracy for grain yield with multivariate models and compared grain yield selection responses to different selection strategies. Combining GS into the conventional breeding program and optimizing the breeding strategies would facilitate and fast-track the genetic improvement for safflower.

Genetic parameters are important estimates for quantitative traits. In this study, we nearly doubled the SNPs number compared to our previous study (Zhao et al., 2021). A high marker density could affect the heritability estimation but not impact the prediction accuracy too much (Zhao et al., 2021). To increase the marker density, imputation of SNPs with missing rate up to 80% has been reported in the GS study for ryegrass and soybean (Jarquín et al., 2014; Faville et al., 2018). The estimated variance and heritability *via* the multivariate model in our study showed consistent trends with the univariate GBLUP models (Zhao et al., 2021). With the decomposition of covariance between traits in multivariate model, the h^2^ could be estimated more accurately compared with the univariate model. Therefore, our study indicates that the multivariate models may perform as well as the univariate models in genetic parameters estimation.

Combining the correlated trait with high heritability would improve the prediction accuracy for the low heritability traits by the multivariate model. The correlation between traits could be a key factor determining the multivariate model’s advantage over the univariate model (Montesinos-López et al., 2021). In our study, the higher prediction accuracy for the multivariate models combining GY and PH confirmed that the correlation between traits is important for the success of multivariate models. A study with Sorghum also showed that genomic prediction for GY benefits mainly from using PH as a secondary trait (Velazco et al., 2019). The prediction accuracy increase varied across sites for GY, and the highest increase was at site LR, where the estimated heritability for GY and correlation between PH and GY were both low. This indicated that multivariate models could benefit more for the trait when estimated heritability is low. Up to 60% PA increase for low heritability traits was reported in a lentil GS study when implementing multivariate models (Haile et al., 2020). We also evaluated the prediction accuracy of different cross-validation scenarios. The scenario 2, PH only included in the training set (CV2), didn't improve the PA, indicating that adding information of correlated traits in the prediction dataset was essential for improving the prediction accuracy by the multivariate models. A similar study in barley reported that the correlated traits only included in the training population would not change the PA in validation and performed equivalently to the univariate model (Arojju et al., 2020). Plant height could be measured at the early stage, and high throughput phenotyping technology, such as the UAV-based RGB imaging system, has been reported to be used for plant height in wheat (Volpato et al., 2021). It indicated that early grain yield prediction by combining the high throughput PH measurements to improve the GS breeding efficiency could incorporate into future safflower GS breeding.

With the limited genetic and genomic resources for the safflower, taking advantage of the modern breeding tool, such as GS, could fast-track the genetic improvement of the crop. Tradition safflower breeding program depends on the phenotypic selection of the elite parental lines to initiate the crossing. In our study, we have chosen the top 20% of individuals according to the BLUEs and GEBVs, respectively, and the best gain achieved was 0.7 t/ha, about a 23% yield increase in the optimal site. This indicated the high yield potential for safflower genetic improvement. We expected to find the highest response by PS for within site selection because the selection differential was used to predict selection response in the next-generation. However, the selection responses in PS and GS were very similar, which could be associated with the fact that most of the top 20% of the accessions with the highest phenotypes had the highest GEBVs as well (Supplementary Table S4). Selection for rust resistance in wheat had similar observations that gain from GS was equal to PS when comparing the gain by two cycles of GS and one cycle of PS (Rutkoski et al., 2015). Bernardo (2020) recommended that selection based on breeding ‘lines’ performance (BLUEs) instead of GEBVs would be sufficient enough if the breeding goal is to pyramiding the elite alleles. However, with GS, selection decisions could be made in an early stage, for example, selection for grain yield before harvest. And when the breeding cycle increases, GS would benefit the breeding program significantly by reducing the breeding cycle. Those lines with high yield and high GEBVs in the safflower collection could be selected directly to achieve yield gain. Selection in the stressed environment, especially for drought resistance, has been reported to improve the selection in optimal conditions (Das et al., 2021; Kumar et al., 2021). However, we observed the selection in the RF and LR sites leads to the compromised gain for GY in IR. Although the gains from different strategies were high at the selected sites, the gap between the optimal and stressed environments was significant. The decreased genetic variation in the stress sites could be the reason for the lower selection response for GY in those sites (Xu et al., 2017). Falconer (1952) mentioned that the correlation between environments could impact the selection responses. In other words, g × E could reduce the rate of genetic gain achieved by breeding. The combined g × EGS selection increased gain slightly in RF and LR, indicating that combining g × E into the prediction model could benefit GS selection. Studies combined multi-environment trials in the genomic prediction model showed similar results (Gill et al., 2021; Khanna et al., 2022). In safflower, using suitable GS models for an optimized breeding pipeline, which selects genotypes that suit environments or genotypes with compromised performance but robust in all conditions, is essential.

Selection indices have been used efficiently for multi-trait breeding programs for animals and crops (Smith, 1936; Hazel, 1943). If selections were based mainly on GY, we observed that OL had limited positive gain in the optimal site and negative gains in stressed sites (Supplementary Table S2). The index selection achieved a high gain for OL and GY although the gain for GY was compromised. The loss of the gain for GY could be compensated by high OL, which would bring high economic values for the farmer. A study in Sorghum also indicated that GS with the optimal index selection to improve biofuel traits is the most promising strategy (Habyarimana et al., 2020a). An optimal breeding framework incorporating index selection has been proposed for different crops (Marulanda et al., 2016; Akdemir et al., 2019). As a minor crop, safflower faces similar challenges as other orphan crops, such as limited resources and limited research funds. GS with selection index is the fast way to pyramid elite alleles into the future safflower varieties. The optimal selection index for safflower GY and OL still requires further research. More information, e.g., biotic and abiotic tolerance, early vigour, seed protein content, etc., could be combined into the selection index. Further validation of different selection strategies, which would facilitate the optimization of breeding strategies by simulation, would also be required.

## Conclusion

In summary, we estimated the heritability and genetic correlations as well as selection responses to different selection indices for a diverse safflower Genebank collection. We achieved higher genetic prediction accuracy for grain yield by combining the correlated trait into the multivariate model. High selection responses for GY were achieved at the selected sites with both PS and GS, but the gains varied across the sites. Combining the g×E model with the equal weighted selection index GS model could simultaneously improve GY and OL in all studied sites.

## Data Availability

The phenotypic dataset and the genotype dataset supporting the conclusions of this article can be found in the Supplementary files. Further inquiries can be directed to the corresponding author/s. The data can also be found here: https://doi.org/10.1002/tpg2.20064.

## References

[B1] Akdemir D. Beavis W. Fritsche-Neto R. Singh A. K. Isidro-Sánchez J. (2019). Multi-objective optimized genomic breeding strategies for sustainable food improvement. Heredity 122, 672–683. 10.1038/s41437-018-0147-1 30262841 PMC6461918

[B2] Arojju S. K. Cao M. Trolove M. Barrett B. A. Inch C. Eady C. (2020). Multi-trait genomic prediction improves predictive ability for dry matter yield and water-soluble carbohydrates in perennial ryegrass. Front. Plant Sci. 11, 1197. 10.3389/fpls.2020.01197 32849742 PMC7426495

[B3] Bernardo R. (2020). Reinventing quantitative genetics for plant breeding: Something old, something new, something borrowed, something BLUE. Heredity 125, 375–385. 10.1038/s41437-020-0312-1 32296132 PMC7784685

[B4] Beyene Y. Semagn K. Mugo S. Tarekegne A. Babu R. Meisel B. (2015). Genetic gains in grain yield through genomic selection in eight Bi-parental maize populations under drought stress. Crop Sci. 55, 154–163. 10.2135/cropsci2014.07.0460

[B5] Bhatta M. Gutierrez L. Cammarota L. Cardozo F. German S. Gomez-Guerrero B. (2020). Multi-trait genomic prediction model increased the predictive ability for agronomic and malting quality traits in barley (hordeum vulgare L.). G3 (Bethesda) 10, 1113–1124. 10.1534/g3.119.400968 31974097 PMC7056970

[B6] Ceron-Rojas J. J. Crossa J. Arief V. N. Basford K. Rutkoski J. Jarquín D. (2015). A genomic selection index applied to simulated and real data. G3 (Bethesda) 5, 2155–2164. 10.1534/g3.115.019869 26290571 PMC4592997

[B7] Das R. R. Vinayan M. T. Seetharam K. Patel M. Phagna R. K. Singh S. B. (2021). Genetic gains with genomic versus phenotypic selection for drought and waterlogging tolerance in tropical maize (Zea mays L.). Crop J. 9, 1438–1448. 10.1016/j.cj.2021.03.012 PMC12806969 33217198

[B8] De Los Campos G. Hickey J. M. Pong-Wong R. Daetwyler H. D. Calus M. P. (2013). Whole-genome regression and prediction methods applied to plant and animal breeding. Genetics 193, 327–345. 10.1534/genetics.112.143313 22745228 PMC3567727

[B9] Dekkers J. C. M. (2007). Prediction of response to marker-assisted and genomic selection using selection index theory. J. Animal Breed. Genet. 124, 331–341. 10.1111/j.1439-0388.2007.00701.x 18076470

[B10] Falconer D. S. Mackay T. F. C. (1996). Introduction to quantitative genetics. 4th Edition. New York: Longman Group Ltd. (Essex)/John Wiley & Sons, Inc.

[B11] Falconer D. S. (1952). The problem of environment and selection. Am. Nat. 86, 293–298. 10.1086/281736

[B12] FAO (2020). faostat/en. Available: https://www.fao.org/faostat/en/#data/QCL .

[B13] Faville M. J. Ganesh S. Cao M. Jahufer M. Z. Z. Bilton T. P. Easton H. S. (2018). Predictive ability of genomic selection models in a multi-population perennial ryegrass training set using genotyping-by-sequencing. Theor. Appl. Genet. 131, 703–720. 10.1007/s00122-017-3030-1 29264625 PMC5814531

[B14] Gill H. S. Halder J. Zhang J. Brar N. K. Rai T. S. Hall C. (2021). Multi-trait multi-environment genomic prediction of agronomic traits in advanced breeding lines of winter wheat. Front. Plant Sci. 12, 709545. 10.3389/fpls.2021.709545 34490011 PMC8416538

[B15] Gilmour A. R. Gogel B. J. Cullis B. R. Welham S. J. Thompson R. (2015). ASReml user guide release 4.1 functional specification. UK: VSN International Ltd, Hemel Hempstead, HP1 1ES.

[B16] Habyarimana E. Lopez-Cruz M. Baloch F. S. (2020b). Genomic selection for optimum index with dry biomass yield, Dry Mass Fraction of Fresh Material, and Plant Height in Biomass Sorghum. Genes (Basel) 11, 61. 10.3390/genes11010061 31948110 PMC7017155

[B17] Habyarimana E. Lopez-Cruz M. Baloch F. S. (2020a). Genomic selection for optimum index with dry biomass yield, dry mass fraction of fresh material, and plant height in biomass Sorghum. Genes 11, 61. 10.3390/genes11010061 31948110 PMC7017155

[B18] Haile T. A. Heidecker T. Wright D. Neupane S. Ramsay L. Vandenberg A. (2020). Genomic selection for lentil breeding: Empirical evidence. Plant Genome 13, e20002. 10.1002/tpg2.20002 33016638 PMC12807041

[B19] Hazel L. N. (1943). The genetic basis for constructing selection indexes. Genetics 28, 476–490. 10.1093/genetics/28.6.476 17247099 PMC1209225

[B20] Heffner E. L. Sorrells M. E. Jannink J.-L. (2009). Genomic selection for crop improvement. Crop Sci. 49, 1–12. 10.2135/cropsci2008.08.0512

[B21] Jarquín D. Kocak K. Posadas L. Hyma K. Jedlicka J. Graef G. (2014). Genotyping by sequencing for genomic prediction in a soybean breeding population. BMC Genomics 15, 740. 10.1186/1471-2164-15-740 25174348 PMC4176594

[B22] Jia Y. Jannink J. L. (2012). Multiple-trait genomic selection methods increase genetic value prediction accuracy. Genetics 192, 1513–1522. 10.1534/genetics.112.144246 23086217 PMC3512156

[B23] Jochinke D. Wachsmann N. Potter T. Norton R. (2008). Growing safflower in Australia: Part 1 - history, experiences and current constraints on production. Australia: The 7th international safflower conference Waga Wagga.

[B24] Khalid N. Khan R. S. Hussain M. I. Farooq M. Ahmad A. Ahmed I. (2017). A comprehensive characterisation of safflower oil for its potential applications as a bioactive food ingredient - a review. Trends Food Sci. Technol. 66, 176–186. 10.1016/j.tifs.2017.06.009

[B25] Khanna A. Anumalla M. Catolos M. Bartholomé J. Fritsche-Neto R. Platten J. D. (2022). Genetic trends estimation in IRRIs rice drought breeding program and identification of high yielding drought-tolerant lines. Rice (N Y) 15, 14. 10.1186/s12284-022-00559-3 35247120 PMC8898209

[B26] Kumar A. Raman A. Yadav S. Verulkar S. B. Mandal N. P. Singh O. N. (2021). Genetic gain for rice yield in rainfed environments in India. Field Crops Res. 260, 107977. 10.1016/j.fcr.2020.107977 33390645 PMC7722510

[B27] Li D. Mündel H. H. (1996). Safflower, Carthamus tinctorius L. promoting the conservation and use of underutilized and neglected crops 7. Rome: Italy, Institute of Plant Genetics and Crop Plant Research, Gatersleben/International Plant Genetic Resources Institute.

[B28] Lin Z. Cogan N. O. I. Pembleton L. W. Spangenberg G. C. Forster J. W. Hayes B. J. (2016). Genetic gain and inbreeding from genomic selection in a simulated commercial breeding program for perennial ryegrass. Plant Genome 9. 10.3835/plantgenome2015.06.0046 27898764

[B29] Lin Z. Hayes B. J. Daetwyler H. D. (2014). Genomic selection in crops, trees and forages: A review. Crop Pasture Sci. 65, 1177–1191. 10.1071/cp13363

[B30] Lozada D. N. Ward B. P. Carter A. H. (2020). Gains through selection for grain yield in a winter wheat breeding program. PLOS ONE 15, e0221603. 10.1371/journal.pone.0221603 32343696 PMC7188280

[B31] Marulanda J. J. Mi X. Utz H. F. Melchinger A. E. Würschum T. Longin C. F. H. (2021). Optimum breeding strategies using genomic and phenotypic selection for the simultaneous improvement of two traits. Theor. Appl. Genet. 134, 4025–4042. 10.1007/s00122-021-03945-5 34618174 PMC8580912

[B32] Marulanda J. Mi X. Melchinger A. Xu J.-L. Würschum T. Longin F. (2016). Optimum breeding strategies using genomic selection for hybrid breeding in wheat, maize, rye, barley, rice and triticale. Theor. Appl. Genet. 129, 1901–1913. 10.1007/s00122-016-2748-5 27389871

[B33] Meuwissen T. Hayes B. Goddard M. (2013). Accelerating improvement of livestock with genomic selection. Annu. Rev. Animal Biosci. 1, 221–237. 10.1146/annurev-animal-031412-103705 25387018

[B34] Meuwissen T. H. Hayes B. J. Goddard M. E. (2001). Prediction of total genetic value using genome-wide dense marker maps. Genetics 157, 1819–1829. 10.1093/genetics/157.4.1819 11290733 PMC1461589

[B35] Michel S. Löschenberger F. Ametz C. Pachler B. Sparry E. Bürstmayr H. (2019). Simultaneous selection for grain yield and protein content in genomics-assisted wheat breeding. Theor. Appl. Genet. 132, 1745–1760. 10.1007/s00122-019-03312-5 30810763 PMC6531418

[B36] Money D. Gardner K. Migicovsky Z. Schwaninger H. Zhong G. Y. Myles S. (2015). LinkImpute: Fast and accurate genotype imputation for nonmodel organisms. G3 5, 2383–2390. 10.1534/g3.115.021667 26377960 PMC4632058

[B37] Montesinos-López A. Runcie D. E. Ibba M. I. Pérez-Rodríguez P. Montesinos-López O. A. Crespo L. A. (2021). Multi-trait genomic-enabled prediction enhances accuracy in multi-year wheat breeding trials. G3 Genes|Genomes|Genetics 11, jkab270. 10.1093/g3journal/jkab270 34568924 PMC8496321

[B38] Rutkoski J. Poland J. Mondal S. Autrique E. Pérez L. G. Crossa J. (2016). Canopy temperature and vegetation indices from high-throughput phenotyping improve accuracy of pedigree and genomic selection for grain yield in wheat. G3 Genes|Genomes|Genetics 6, 2799–2808. 10.1534/g3.116.032888 27402362 PMC5015937

[B39] Rutkoski J. Singh R. P. Huerta-Espino J. Bhavani S. Poland J. Jannink J. L. (2015). Genetic gain from phenotypic and genomic selection for quantitative resistance to stem rust of wheat. Plant Genome 8, eplantgenome2014. 10.3835/plantgenome2014.10.0074 33228306

[B40] Schaeffer L. R. (2006). Strategy for applying genome-wide selection in dairy cattle. J. Anim. Breed. Genet. 123, 218–223. 10.1111/j.1439-0388.2006.00595.x 16882088

[B41] Smith H. F. (1936). A discriminant function for plant selection. Ann. Eugen. 7, 240–250. 10.1111/j.1469-1809.1936.tb02143.x

[B42] Sun J. Poland J. A. Mondal S. Crossa J. Juliana P. Singh R. P. (2019). High-throughput phenotyping platforms enhance genomic selection for wheat grain yield across populations and cycles in early stage. Theor. Appl. Genet. 132, 1705–1720. 10.1007/s00122-019-03309-0 30778634

[B43] Velazco J. G. Jordan D. R. Mace E. S. Hunt C. H. Malosetti M. Van Eeuwijk F. A. (2019). Genomic prediction of grain yield and drought-adaptation capacity in Sorghum is enhanced by multi-trait analysis. Front. Plant Sci. 10, 997. 10.3389/fpls.2019.00997 31417601 PMC6685296

[B44] Volpato L. Pinto F. González-Pérez L. Thompson I. G. Borém A. Reynolds M. (2021). High throughput field phenotyping for plant height using UAV-based RGB imagery in wheat breeding lines: Feasibility and validation. Front. Plant Sci. 12, 591587. 10.3389/fpls.2021.591587 33664755 PMC7921806

[B45] Woolliams J. A. Berg P. Dagnachew B. S. Meuwissen T. H. E. (2015). Genetic contributions and their optimization. J. Animal Breed. Genet. 132, 89–99. 10.1111/jbg.12148 25823835

[B46] Xu Y. Li P. Zou C. Lu Y. Xie C. Zhang X. (2017). Enhancing genetic gain in the era of molecular breeding. J. Exp. Bot. 68, 2641–2666. 10.1093/jxb/erx135 28830098

[B47] Zhao H. Li Y. Petkowski J. Kant S. Hayden M. J. Daetwyler H. D. (2021). Genomic prediction and genomic heritability of grain yield and its related traits in a safflower genebank collection. Plant Genome 14, e20064. 10.1002/tpg2.20064 33140563 PMC12807229

[B48] Zhao H. Pandey B. R. Khansefid M. Khahrood H. V. Sudheesh S. Joshi S. (2022a). Combining NDVI and bacterial blight score to predict grain yield in field pea. Front. Plant Sci. 13, 923381. 10.3389/fpls.2022.923381 35837454 PMC9274273

[B49] Zhao H. Savin K. W. Li Y. Breen E. J. Maharjan P. Tibbits J. F. (2022b). Genome-wide association studies dissect the G × E interaction for agronomic traits in a worldwide collection of safflowers (Carthamus tinctorius L.). Mol. Breed. 42, 24. 10.1007/s11032-022-01295-8 PMC10248593 37309464

[B50] Zohary D. (1999). Monophyletic vs. polyphyletic origin of the crops on which agriculture was founded in the Near East. Genet. Resour. Crop Evol. 46, 133–142. 10.1023/a:1008692912820

