# Supplementary material for: Genomic prediction and selection response for grain yield in safflower
Source: Front Genet. 2023 Mar 27;14:1129433. doi: 10.3389/fgene.2023.1129433 (PMC10083426; doi:10.3389/fgene.2023.1129433)
Supplement: Supplementary file 2 [file Table1.DOCX]

**Genomic prediction and selection response for grain yield in safflower**

Huanhuan Zhao1,2*, Zibei Lin2, Majid Khansefid1,2, Josquin F. Tibbits2, Matthew J. Hayden1,2

1 School of Applied Systems Biology, La Trobe University, Bundoora, VIC 3083, Australia

2 Agriculture Victoria, AgriBio, Centre for AgriBioscience, Bundoora, VIC 3083, Australia

* Corresponding author Huanhuan Zhao, huan.zhao@agriculture.vic.gov.au


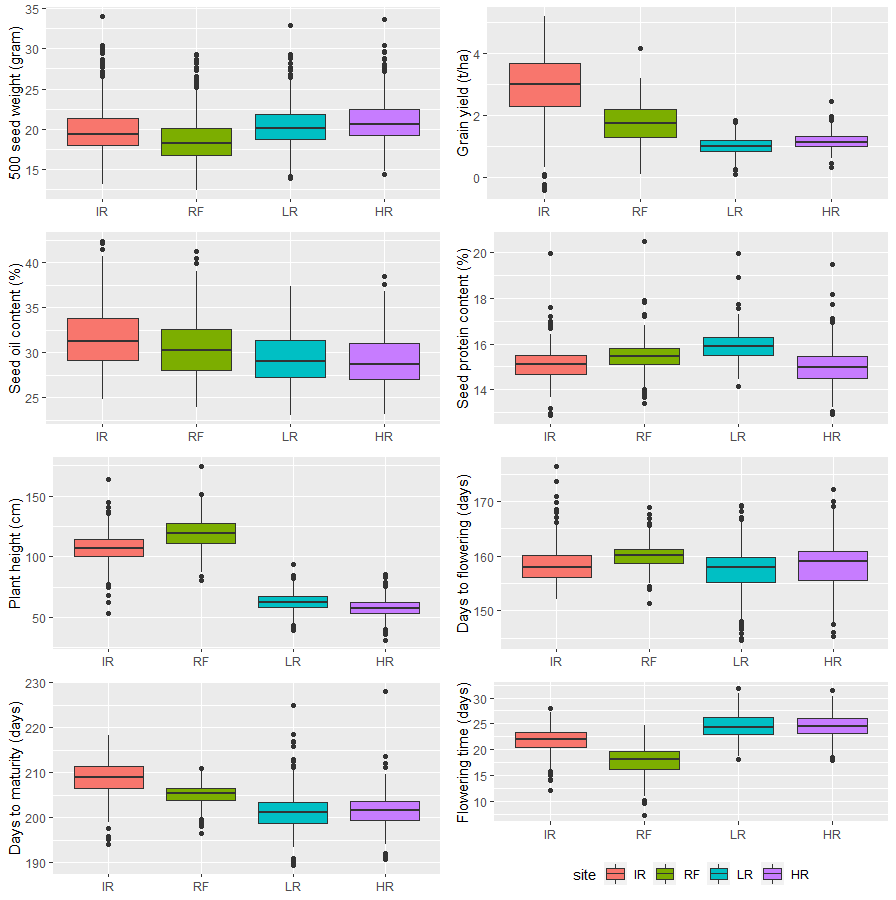


Supplementary Fig s1. Boxplot of safflower traits’ BLUEs in four sites.


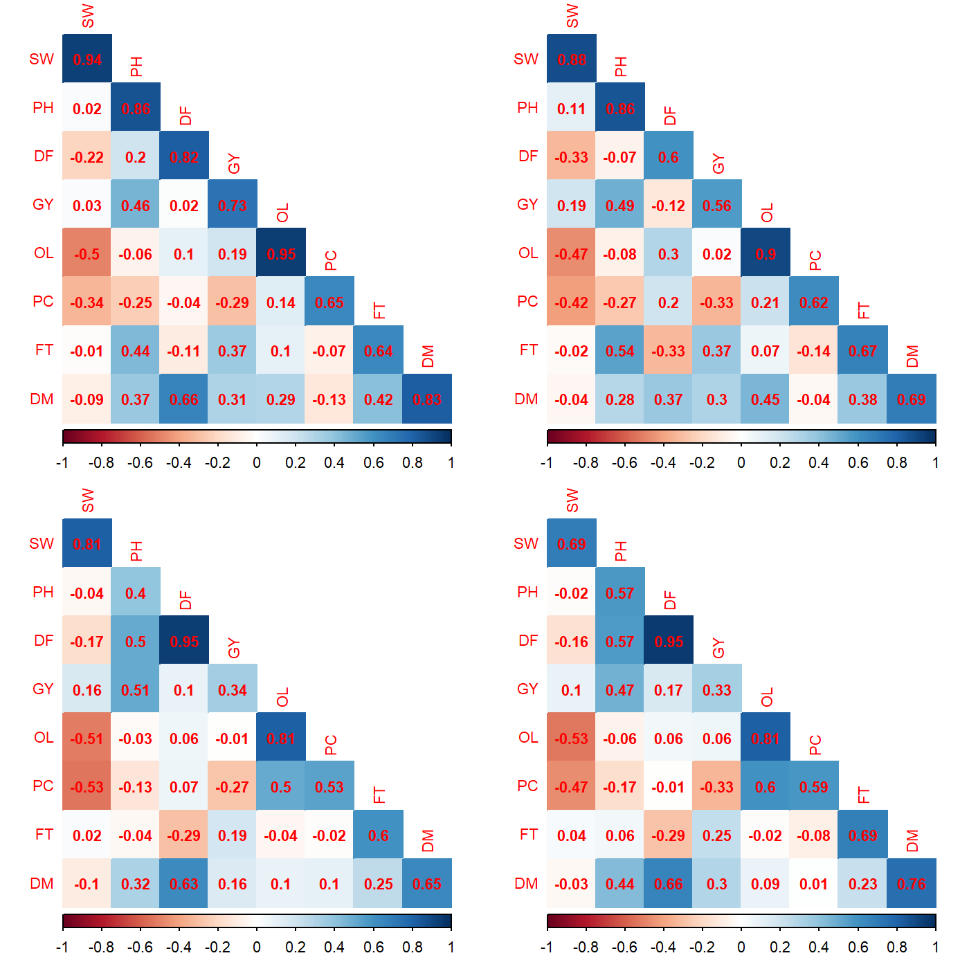


Supplementary Fig s2. The phenotypic correlation (r_p_, lower triangle) estimated by multivariate models among pairwise safflower traits in four field sites (clockwise, IR,RF,HR, IR). Color ranged from dark orange to dark blue indicates the correlation r_p_ from -1 to 1.
